# Research Progress on the Role of RNA m6A Modification in Glial Cells in the Regulation of Neurological Diseases

**DOI:** 10.3390/biom12081158

**Published:** 2022-08-21

**Authors:** Siyi You, Xiaojuan Su, Junjie Ying, Shiping Li, Yi Qu, Dezhi Mu

**Affiliations:** 1 Department of Pediatrics, West China Second University Hospital, Sichuan University, Chengdu 610041, China; 2 Key Laboratory of Birth Defects and Related Diseases of Women and Children (Sichuan University), Ministry of Education, NHC Key Laboratory of Chronobiology, Sichuan University, Chengdu 610041, China

**Keywords:** RNA m6A modification, microglia, astrocyte, oligodendrocyte, neurological disease

## Abstract

Glial cells are the most abundant and widely distributed cells that maintain cerebral homeostasis in the central nervous system. They mainly include microglia, astrocytes, and the oligodendrocyte lineage cells. Moreover, glial cells may induce pathological changes, such as inflammatory responses, demyelination, and disruption of the blood–brain barrier, to regulate the occurrence and development of neurological diseases through various molecular mechanisms. Furthermore, RNA m6A modifications are involved in various pathological processes associated with glial cells. In this review, the roles of glial cells in physiological and pathological states, as well as advances in understanding the mechanisms by which glial cells regulate neurological diseases under RNA m6A modification, are summarized, hoping to provide new perspectives on the deeper mechanisms and potential therapeutic targets for neurological diseases.

## 1. Introduction

Glial cells are the most abundant and widely distributed cells that maintain cerebral homeostasis in a temporal and spatial dependent manner in the central nervous system (CNS). They include the microglia, astrocytes, and oligodendrocyte lineage cells [1]. Studies have suggested that the structures and functions of glial cells change in response to the stimuli encountered, which in turn induces pathological changes, such as inflammation, demyelination, and disruption of the blood–brain barrier (BBB), ultimately leading to the occurrence and development of neurological diseases through various molecular mechanisms [2]. Therefore, the mechanism by which glial cells respond to stimuli plays a critical role in determining the fate of cells and diseases, as glial cells are involved in the disease process. Cells usually depend on a complex molecular regulation process in response to stimuli, which is mainly achieved by the regulation of intracellular biological macromolecules, especially RNA. 

**Figure 1:**
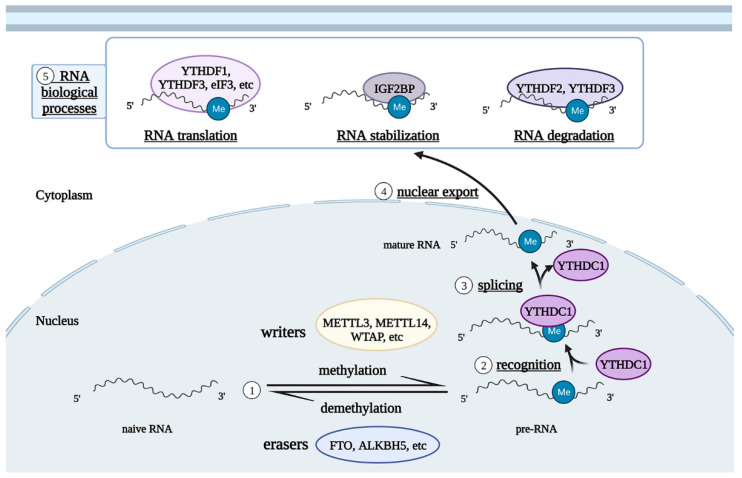
Illustration of RNA m6A modification patterns. RNAs are reversibly modified by m6A through writers and erasers, and are spliced, exported to the cytoplasm, translated, degraded, or stabilized by readers. Abbreviations: METTL3, methyltransferase-like 3; METTL14, methyltransferase-like 14; WTAP, Wilm’s tumor 1-associating protein; FTO, fat mass and obesity-associated protein; ALKBH5, AlkB homolog 5; YTHDC1, YTH domain containing 1; YTHDF1/2/3, YTH domain family 1/2/3; IGF2BP, insulin-like factor-2 mRNA binding protein; eIF3, eukaryotic initiation factor 3.

Epitranscriptomic modification, including additions of N6-methyladenosine (m6A) and C5-methylcytidine (m5C), is a key molecular regulation mechanism that affects cell functions by regulating gene expression patterns, thereby mediating the occurrence and development of diseases [3]. Among these, m6A epigenome modification is the most abundant post-transcriptional modification of mRNA, particularly in the brain of mammals [4]. It refers to the methylation of the 6th N atom of adenine and is particularly frequent in highly-conserved regions with an RRACH consensus sequence (R = G or A; H = U, A, or C) that is near the stop codon of coding sequence (CDS) and 3′ untranslated region (3′ UTR) and within internal long exons. m6A modification is dynamically regulated by a series of enzymes, which can be divided into three types, according to their functions: writers, erasers, and readers [5]. Writers are a group of methyltransferases that recognize the RRACH sequences directly through the methyltransferase domain (MTD) or function, by stabilizing methyltransferase complexes and recruiting other methyltransferases to transfer the methyl from S-adenosylmethionine (SAM) at corresponding sites of mRNA, rRNA, and other ncRNAs. Writers contain methyltransferase-like 3 (METTL3), methyltransferase-like 14 (METTL14), Wilm’s tumor 1-associating protein (WTAP), RNA binding motif protein 15/15B (RBM15/15B), vir-like m6A methyltransferase associated (VIRMA) subunits, zinc finger CCCH domain-containing protein 13 (ZC3H13), and Cbl proto-oncogene like 1 (CBLL1) etc. [6,7,8,9]. Erasers are mainly demethyltransferases, of which fat mass and obesity-associated protein (FTO) and AlkB homolog 5 (ALKBH5) are the most important components, belonging to the α-ketoglutarate (α-KG)-dependent dioxygenase family and functioning by catalyzing m6A removal [10]. After the modification of writers and erasers, the final functional exertion, fate, and destination of m6A-modified RNA depend on the combination of readers (e.g., YTH domain containing 1/2 (YTHDC1/2), YTH domain family 1/2/3 (YTHDF1/2/3), heterogeneous nuclear ribonuclease (hnRNP) family hnRNPA2/B1 and hnRNPC/G, insulin-like factor-2 mRNA binding protein (IGF2BP), eukaryotic initiation factor 3 (eIF3), and fragile X mental retarding protein (FMRP)), which have unique m6A recognition domains at a specific time and space to alter biological processes, including splicing, nuclear export, translation, stabilization, and degradation [11,12,13,14] (Figure 1).

Several studies have recently confirmed that RNA m6A modification is involved in the regulation of glial cell physiology and pathology, which mediates the development of neurological systems and neurological diseases. However, reviews focusing on summarizing and clarifying the relationship between RNA m6A-modified glial cells and neurological diseases are lacking. Therefore, the aim of this review was to systematically review the research progress in RNA m6A modification of glial cells for the regulation of neurological diseases. For this review, we aimed to (1) summarize and analyze the roles and functions of glial cells in different physiological and pathological states; (2) clarify the roles of RNA m6A modification of glial cells in mediating the occurrence or development of specific neurological diseases and indicate the detailed mechanism of the RNA m6A modification of glial cells; and (3) discuss and analyze the current research status and future directions of RNA m6A modification in glial cells for regulating neurological diseases, which may provide a reference for researchers in this field. 

## 2. Roles of Glial Cells in Physiological and Pathological States

### 2.1. Roles of Microglia in Physiological and Pathological States

**Figure 2:**
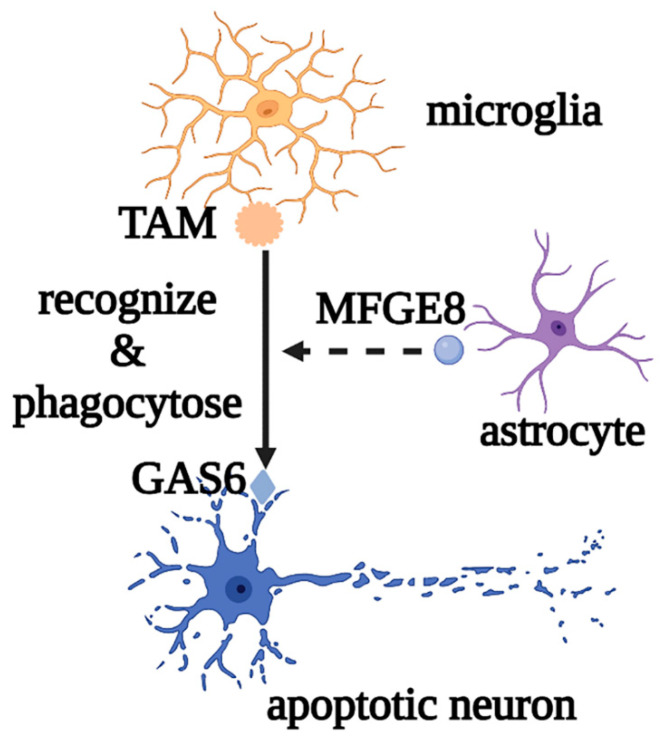
Roles of microglia in phagocytosing apoptotic neurons. Microglia mediate the phagocytosis of apoptotic neurons through the recognition of TAM and GAS6, as well as the auxiliary effect of MFGE8. Abbreviations: GAS6, growth arrest-specific protein 6; TAM, tyro3, axl, and mer; MFGE8, milk fat globulin epidermal growth factor 8.

Microglia are the most important innate immune cells in the CNS. They originate from early yolk sac erythromyeloid progenitors and migrate into the neural tube, where they proliferate and colonize the parenchyma before angiogenesis, as the first glial cells to migrate into the CNS. Macrophages and dendritic cells from the non-cerebral parenchyma (such as the meninges, choroid plexus, and perivascular space) can enter the parenchyma and transform into microglia during embryonic development [15,16]. Microglial development and maintenance depend on the binding of colony stimulating factor 1 receptor (CSF1R) with colony stimulating factor 1 (CSF1) and IL-34, secreted by neurons and glial cells, and microglia fully mature under the influence of TGF-β secreted by both cells [17]. Under normal physiological conditions, microglia, also called “resting microglia,” show the typical branching morphology, with small cell bodies and thin protrusions, and continuously regulate changes in the brain microenvironment by engulfing apoptotic or excess cells and regulating synaptic plasticity and neurogenesis, to maintain the homeostasis of the brain environment. For example, when apoptotic neurons are phosphatidylserine-labeled by growth arrest-specific protein 6 (GAS6), microglia can recognize and phagocytose neurons via the tyro3, axl, and mer (TAM) and milk fat globulin epidermal growth factor 8 (MFGE8) released by astrocytes [18,19] (Figure 2).

**Figure 3:**
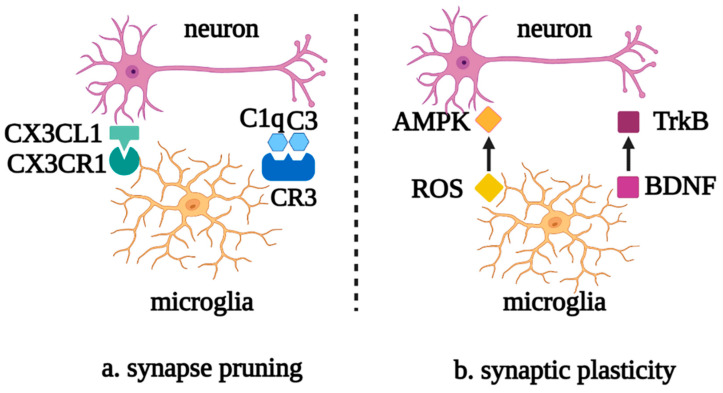
Roles of microglia in neuronal synapse pruning and plasticity. (**a**) Microglia mediate the neuronal synapse pruning via the complement cascade and the recognition of CX3CR1 and CX3CL1. (**b**) Microglia mediate synaptic plasticity via the mutual effects of ROS and AMPK, as well as BDNF and TrkB. Abbreviations: CX3CL1, C-X3-C motif chemokine ligand 1; CX3CR1, C-X3-C motif chemokine receptor 1; C1q, complement 1q; C3, complement 3; CR3, complement receptor 3; AMPK, AMP-activated protein kinase; ROS, reactive oxygen species; TrkB, tyrosine kinase receptor B; BDNF, brain-derived neurotrophic factor.

Microglia also perform synaptic pruning through the direct interaction of the chemokine receptor, CX3CR1, with the transmembrane protein, CX3CL1, on the neuron surface or via the activation of the complement cascade C1q/C3/C3R. Meanwhile, synaptic activity and plasticity can be regulated by reactive oxygen species (ROS), which are secreted by microglia, downregulating the expression of AMPK receptors and brain-derived neurotrophic factor (BDNF)-binding TrkB receptors [20,21] (Figure 3). 

Microglia can also secrete insulin-like growth factor 1 (IGF-1), IL-4, and nitric oxide (NO), promoting the differentiation of neural progenitor cells (NPCs) into oligodendrocytes (OLs) and regulating neurogenesis [22]. 

**Figure 4:**
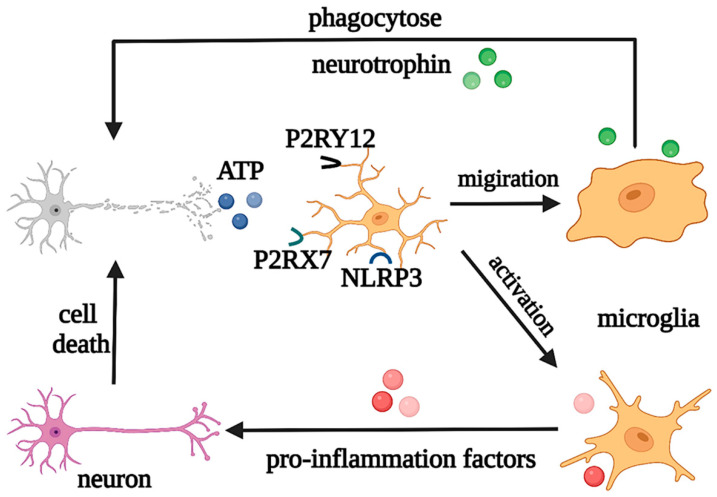
Regulation of microglia under pathological states. Under pathological states, microglia play neuroprotective and neurotoxic roles. Abbreviations: ATP, adenosine triphosphate; P2RY12, purinergic receptor P2Y12; P2RX7, purinergic receptor P2X7; NLRP3, NOD-like receptor (NLR) family pyrin domain-containing protein 3.

In addition, as neuroinflammation is one of the common features of CNS pathology, microglia are involved in the regulation of the pathological processes in both acute inflammation in acute injury and chronic inflammation in neurodegeneration [23,24]. For instance, when induced by Toll-like receptors (TLRs) and other stimuli, microglia are activated with retracted and thickened protrusions and enlarged cell bodies, morphed into short and coarse branching or an ameboid shape, and polarized into M1 and M2 phenotypes according to the pathological damage conditions, time, severity of injury, age, and microenvironment. They participate in antigen presentation, the release of pro-inflammatory/anti-inflammatory cytokines, and the phagocytosis process of necrotic cells. M1 microglia with short and thick branches secrete pro-inflammatory cytokines, such as IL-1β, IL-6, tumor necrosis factor alpha (TNF-α), and neurotoxic substances ROS and NO, which are involved in worsening clinical prognosis in most cases [25]. On the other hand, M2 microglia have a typical anti-inflammatory phenotype and ameboid morphology and secrete IL-10, TGF- β, IGF-1, fibroblast growth factor (FGF), CSF1, and neurotrophic factors, to suppress inflammation, engage in regeneration, or mediate phagocytosis [26]. In the early stage of inflammation, the excessive adenosine triphosphate (ATP) and glutamate released by damaged neurons activate N-methyl-D-aspartate (NMDA) receptors, which induce excitatory neurotoxicity and lead to neuronal death [27]. However, ATP can also cause negative feedback, with the activation of the microglial purinergic receptor P2Y12, inducing the migration of microglia to the injured areas to promote nerve regeneration [28]. At the same time, in pathological states, microglia can also engulf pathologically folded proteins, such as tau protein, and myelin sheath fragments, which limits disease progression in the early stage. However, with further development of inflammation, ATP accumulation promotes the activation of the microglial purinergic receptor P2X7 (P2RX7) and NOD-like receptor (NLR) family pyrin domain-containing protein 3 (NLRP3), resulting in the release of excessive pro-inflammatory and neurotoxic factors, which cause phagocytosis dysfunction and brain tissue damage [29]. Moreover, microglia can produce prostaglandin D2, which acts on the prostaglandin receptor DP1 of astrocytes, to induce demyelination [16]. It can be seen that the morphological changes of microglia in the nervous system are accompanied by corresponding changes in their phenotypes and functions, and each phenotype plays a unique role in the occurrence and development of neurological diseases (Figure 4).

### 2.2. Roles of Astrocytes in Physiological and Pathological States 

**Figure 5:**
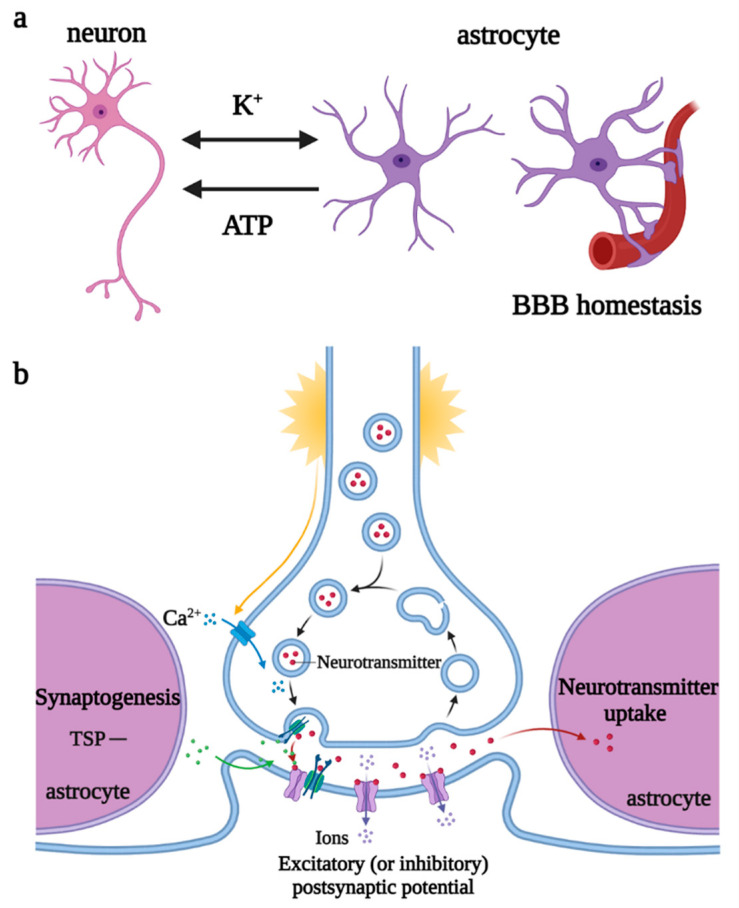
Regulations of astrocytes under physiological states. (**a**) Astrocytes participate in K^+^ balance, release ATP to regulate neuronal excitability, and maintain BBB homeostasis. (**b**) Astrocytes promote synaptogenesis and participate in the regulation of neurotransmitter release and recycling. Abbreviations: ATP, adenosine triphosphate; BBB, blood–brain barrier; TSP, thrombospondin.

Astrocytes are the most widely distributed and numerous glial cells in the mammalian brain. They originate from NPCs and radial glial cells (RGCs) in the subventricular zone, express glial fibrillary acidic protein (GFAP), a specific marker for astrocytes in some regions of the brain, and migrate to the cortical layers, to fill the neuronal space, supporting and dividing neurons. Astrocytes are involved in the regulation of neurotransmitter release and recycling, K^+^ and Ca^2+^ balance, synaptogenesis, plasticity maintenance, and BBB homeostasis [30]. When stimulated by the K^+^ and neurotransmitters released by neurons, astrocytes depolarize and trigger the elevation of [Ca^2+^]_i_ and [K^+^]_i_ and release of vesicle ATP, regulating the excitability of the axon initial segment of excitatory neurons and the conduction velocity of myelinated axons, thereby controlling neuronal energy metabolism and synaptic numbers [31,32] (Figure 5). 

**Figure 6:**
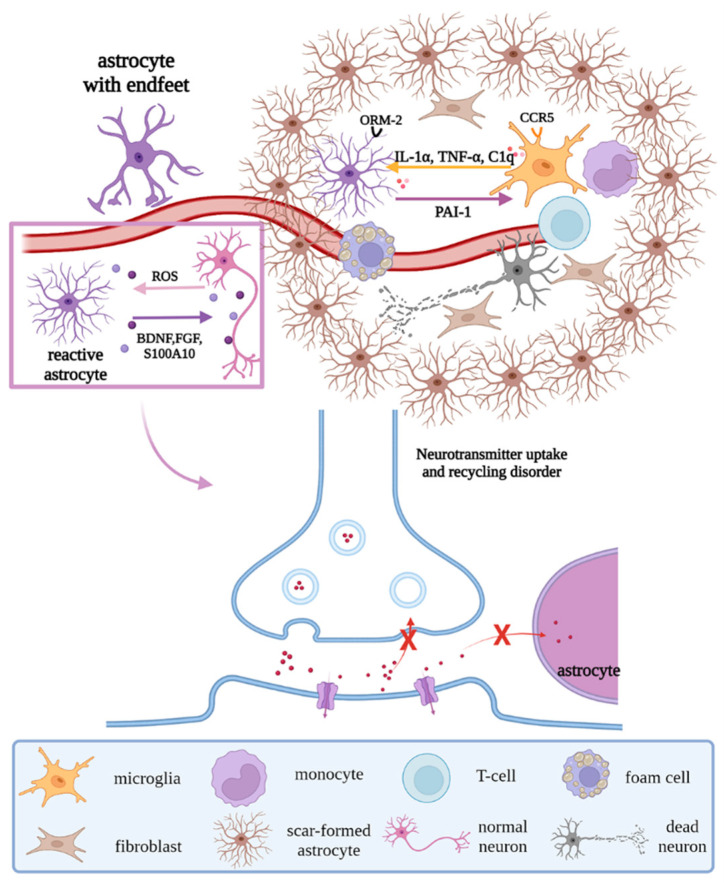
Regulations of astrocytes under pathological states. Under pathological states, reactive astrocytes remove ROS and release neurotrophic factors, to promote the survival of neurons; in addition, their endfeet strip off the vascular surface, resulting in the destruction of the BBB and dysregulation of synaptic release and recycling, with morphological changes. Moreover, under the interaction with microglia and stimulation of factors secreted by microglia, scar-formed astrocytes, peripheral immune cells, and fibroblasts form glial scar. Abbreviations: ROS, reactive oxygen species; BDNF, brain-derived neurotrophic factor; FGF, fibroblast growth factor; S100A10, S100 calcium binding protein A10; ORM-2, orosomucoid-2; CCR5, C-C chemokine receptor type 5; C1q, complement 1q; PAI-1, plasminogen activator inhibitor type 1; “×”, the infeasibility of the approach.

Previous studies have shown that, on the one hand, astrocytes undergo reactive hypertrophy and hyperplasia when stimulated by injury, differentiating into A1 neurotoxic and A2 neuroprotective phenotypes. A1 astrocytes promote the production of pro-inflammatory factors and chemokines through the JAK/STAT and NF-κB pathways, resulting in the dysregulation of synaptic release and recycling and morphological changes of synapses and the formation of glial scars under the stimulation of IL-1α, TNF-α, and C1q secreted by microglia [33,34]. A2 astrocytes can clear ROS and release neurotrophic factors such as BDNF, FGF, nerve growth factor (NGF), and S100 calcium-binding protein A10 (S100A10) to promote neuron survival [35]. On the other hand, dysfunctional ion transport in astrocytes leads to neurotoxic cerebral edema, and the morphologic changes in astrocytes cause the endfeet to detach from the vascular surface, destroying the BBB, which allows peripheral blood T cells and monocytes to infiltrate the brain parenchyma and interact with microglia, to exacerbate the inflammatory response [36,37]. At the same time, astrocyte orosomucoid-2 binds to the C-C chemokine receptor type 5 (CCR5) of microglia to block the CXCL4-CCR5 binding domain, inhibiting microglial activation and migration [38]. Astrocytes regulate microglial phagocytic activity via plasminogen activator inhibitor type 1 (PAI-1) release, the low-density lipoprotein receptor-related protein 1 (LRP-1)/JAK/STAT1 axis, and TLR2/6 [39] (Figure 6). Together, we can conclude that astrocytes can transform their functions through morphological changes, in response to external stimuli, ultimately leading to the further development of neurological diseases.

### 2.3. Roles of Oligodendrocytes in Physiological and Pathological States 

Oligodendrocyte lineage cells are the myelin-forming cells in the CNS, which mainly include the oligodendrocyte progenitor cells (OPCs), pre-OLs, and OLs. Arising from NPCs and subsisting during the process of development, OPCs, also known as NG2 glial cells (whose markers also include oligodendrocyte transcription factor (Olig2) and SRY-box transcription factor 10 (SOX10)), have a bipolar morphology and maintain a highly dynamic balance between proliferation and differentiation. OPCs migrate to myelination sites via the WNT pathway, to crawl or jump along blood vessels, and continuously produce bipolar pre-OLs throughout their life cycle [40,41,42]. When in contact with axons, the bipolar morphology of pre-OLs changes, and pre-OLs differentiate into mature OLs (whose markers include myelin associated glycoprotein (MAG), myelin basic protein (MBP), proteolipid protein (PLP), and 2′, 3′-cyclic nucleotide 3′phosphodiesterase (CNPase)). The cytoplasm of OLs wraps axons and forms a myelin sheath for rapid action potential transmission, owing to the high resistance and low capacitance of the sheath and the existence of the nodes of Ranvier. OLs can also provide nutritional and metabolic support for axons by transporting lactate and ATP [43]. 

**Figure 7:**
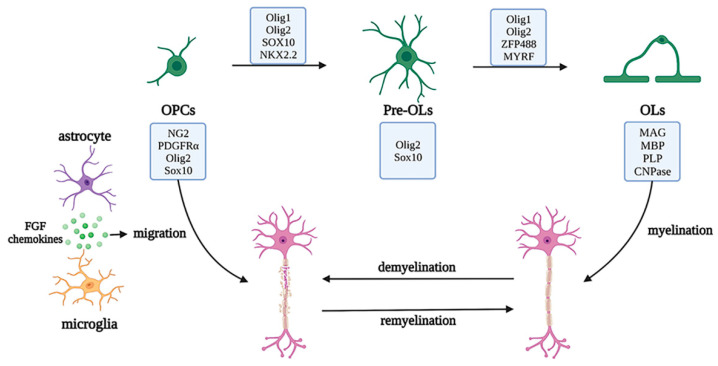
Development of the oligodendrocyte lineage and migration and regeneration under pathological conditions. The oligodendrocyte lineage is composed of the OPCs, pre-OLs, and OLs with unique markers and OLs cytoplasm, which wraps axons to form myelin sheaths. Under pathological conditions, the myelin is damaged, the neurons are demyelinated, and myelin regeneration is initiated by OPC activation and migration to injured sites, with the assistance of astrocytes and microglia and a series of transcription factors. Abbreviations: PDGFRα, platelet derived growth factor receptor alpha; SOX10, SRY-box transcription factor 10; NKX2.2, NK2 homeobox 2; Olig1/2, Oligodendrocyte transcription factor 1/2; ZFP488, zinc finger protein 488; MYRF, myelin regulatory factor; MAG, myelin associated glycoprotein; MBP, myelin basic protein; PLP, proteolipid protein; CNPase, 2′,3′-cyclic nucleotide 3′phosphodiesterase; OPC, oligodendrocyte progenitor cells; OL, oligodendrocytes.

OLs are noted as crucial cells for myelination, and mature OL deficiency may cause axonal loss and demyelination, leading to brain injury, autoimmune diseases, demyelinating or chronic degenerative diseases, and other myelination disorders. Under normal physiological conditions, a deficiency of OLs induces the differentiation of OPCs into new OLs and migration to the lesion sites, under the secretion of factors by microglia and astrocytes, such as BDNF and FGF, to restart the myelination process and cover the injured sites or axons that have not previously been myelinated [44]. During myelin regeneration, the transcription factors required for OPC differentiation, such as NK2 homeobox 2 (NKX2.2), myelin regulatory factor (MYRF), zinc finger protein 488 (ZFP488), Olig1, and Olig2, are highly expressed, to initiate myelinogenesis. However, pathological studies of most demyelinating diseases have shown that OPCs are the main damaged cells, including the obstruction of differentiation of OPCs to OLs (e.g., white matter injury diseases), inhibition of OPC migration to the injured sites (e.g., multiple sclerosis), and ultimately increased OPC apoptosis (e.g., senescence). Therefore, the functions of OL lineage cells change through a variety of molecular mechanisms, further regulating the occurrence and development of various neurological diseases [45,46] (Figure 7).

## 3. Mechanism of RNA m6A-Modified Glial Cells in the Occurrence and Development of Neurological Diseases

**Figure 8:**
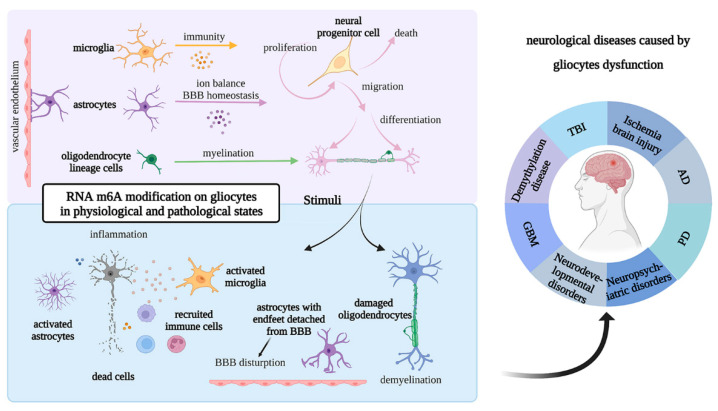
Illustration of the m6A modification of glial cells in physiological and pathological processes. Glial cells contribute to the determination of cell fate—proliferation, differentiation, migration, and death—in the aspects of immunity, ion balance and BBB homeostasis, and myelination. Moreover, glial cell changes induce pathological alterations, including inflammation, demyelination, and disruption of BBB, leading to a series of neurological diseases. All of the structures and functions of glial cells can change via intracellular RNA regulation. Abbreviations: TBI, traumatic brain injury; AD, Alzheimer’s disease; PD, Parkinson’s disease; BBB, blood–brain barrier.

Based on the above summary and analysis of glial cells, they may participate in regulating the occurrence and development of neurological diseases through different molecular mechanisms. Recent studies have shown that RNA m6A is involved in the pathological processes of a variety of neurological diseases, such as Alzheimer’s disease (AD), multiple sclerosis (MS), and depression. However, a summary and analysis of the regulatory mechanism of RNA m6A modification in glial cells in neurological diseases has not yet been reported. Therefore, from the perspective of RNA m6A-modified glial cells engaging in the regulation of neurological diseases, this section summarizes and analyzes the correlation between glial cells and various neurological diseases, including traumatic brain injury (TBI), demyelination diseases, ischemic brain injury, AD, Parkinson’s disease (PD), neuropsychiatric disorders, neurodevelopmental disorders, and glioblastoma (GBM) (Figure 8).

### 3.1. TBI Mediated by RNA m6A-Modified Glial Cells

TBI is an acute brain injury that leads to irreversible neuronal death and axon rupture followed by neuroinflammation, during which activated microglia and astrocytes are polarized into different phenotypes, to secrete neuroprotective or neurotoxic factors. Subsequently, inflammasomes and glial scars are formed, cell fragments are phagocytosed, or BBB permeability is changed to exacerbate or ameliorate injury, under the stimulation of a damage-associated molecular pattern (DAMP). OL dysfunction is also present in demyelinating processes, such as axon swelling, demyelination, and microtubule rupture [47,48]. 

Recent studies have shown that RNA m6A modifications are involved in the development of TBI (Table 1). After its occurrence, the expression levels of METTL14 and FTO were significantly downregulated in the cortical tissues of a controlled cortical injury (CCI) model in rats, without significant changes in the expression of METTL3, WTAP, VIRMA, and ALKBH5, resulting in alterations in the expression levels of m6A-modified RNA and a large number of genes, particularly the downregulation of Bcl-2 expression and upregulation of Dll4 (activating the Notch pathway) and CD14 (reflecting acute inflammation in perivascular and cerebral parenchyma) expression [49]. Although FTO plays an important role in neurological recovery and the maintenance of neurological functions after TBI, the expression of METTL3 and total RNA m6A in hippocampal neurons were downregulated at the earlier stage of CCI in mice, without significant METTL14, FTO, WTAP, and ALKBH5 expression changes, which may be related to impaired cognitive functions [50]. The difference between the two studies might be due to the type of model organisms and time of injury.

### 3.2. Demyelination Diseases Mediated by RNA m6A-Modified Glial Cells

MS is a typical autoimmune demyelination disease that manifests as the first clinically isolated syndrome with motor or sensory problems and brainstem dysfunction. It is characterized by the demyelination of lesions and inflammatory cell infiltration in multiple regions and time points, where OPC differentiation is inadequate [82,83]. Microglia are activated to engulf myelin fragments [84]. Meanwhile, astrocytes recruit lymphocytes, secrete chemokines to recruit OPCs to demyelinated regions, release pro-inflammatory factors, and form glial scars, which induce excitatory toxic damage to the OLs, neuron demyelination, axonal degeneration, and chronic CNS inflammation [85].

Studies have shown that under normal physiological conditions, there are changes in YTHDF2 and METTL14 expression during OL lineage development [51,53]. Moreover, hnRNPA2/B1 is responsible for the transport of myelin basic protein (MBP) mRNA [52]. METTL14 knockdown of OL lineage cells resulted in the inhibition of OL lineage differentiation and decreased myelination. Furthermore, the m6A levels were altered in SHH, ERK/MAPK, PI3K/AKT/mTOR, and other pathways related to OL maturation, which may be associated with abnormal RNA splicing, leading to significantly reduced expression of neurofascin 155 in Ranvier′ s node and abnormal myelin sheath function [51]. Proline rich coiled-coil 2a (Prrc2a) is also involved in the post-transcriptional regulation of Olig2 mRNA, which is associated with OPC differentiation [53]. In addition, FTO binds to axon GAP-43 mRNA, resulting in decreased methylation and increased translation, thereby promoting axon lengthening [54]. In addition, the m6A modification of Olig2 mRNA decreased, leading to a decrease in myelination and cognitive function. Patients with progressive multiple sclerosis (PMS) have lower mRNA m6A levels in the cerebrospinal fluid (CSF) than those with relapsing remitting multiple sclerosis (RRMS), which provides a possible marker for early differentiation between PMS and RRMS [86] (Table 1).

### 3.3. Ischemic Brain Injury Mediated by RNA m6A-Modified Glial Cells

Ischemic brain injury, of which the most common is ischemic stroke, is caused by insufficient blood supply to local or multiple regions. Once this occurs, microglia can accumulate within a few minutes, with the recruitment of immune cells from the circulatory system to the sites of injury. Similarly, microglia are activated and polarized by the A2A adenosine receptor and pattern recognition receptors (PRRs), to secrete pro-inflammatory or neuroprotective mediators and phagocytose cell fragments, with inhibition of the proliferation and differentiation of neural stem cells (NSCs) [87]. In addition, astrocytes are activated under P2Y1 stimulation for dual roles with astrocyte-mediated excitatory amino acid transporter (EAAT) disorder and damage to aquaporin4, leading to glutamate excitatory cytotoxicity, cerebral blood flow disorder, and cerebral edema. Inflammatory factors also inhibit the proliferation and differentiation of cells of the OL lineage, resulting in demyelination and worsening prognosis [88,89].

Classical models of ischemic brain injury can be established by middle cerebral artery occlusion (MCAO) and oxygen and glucose deprivation (OGD). Using rat MCAO and neuron OGD models, several studies have revealed the possible roles of m6A modification. For example, downregulation of ALKBH5 and FTO expression and upregulation of m6A levels, in turn promote the degradation of Bcl-2 mRNA, leading to a downregulation of Bcl-2 expression and neuronal apoptosis [57]. In addition, a study indicated that to induce the death of neurons, METTL3 mediated the increase in m6A modification of lnc-D63785. As competitive endogenous RNA, downregulated lnc-D63785 led to the accumulation of miR-422a and the downregulation of expression of the downstream transcription factors MEF2D (regulating Bcl-w to promote neuronal survival) and MAPKK6 (regulating p38) [90]. In contrast, in the early stage of brain injury, METTL3 upregulated the m6A level of pri-miR-335 and increased its maturation. It specifically downregulated the expression of eukaryotic translation termination factor 1 (Erf1) and promoted the formation of stress granules, which in the ischemic cortex was inversely proportional to the level of apoptosis and the severity of injury. Thus, cell apoptosis was reduced and METTL3 played a neuroprotective role [91]. In addition, YTHDC1 may reduce the stability of phosphatase and tensin homolog (*PTEN*, a tumor suppressor gene that inhibits the downstream PI3K/Akt oncogenic pathway) and downregulate its expression by binding to the 3′ UTR of PTEN mRNA, thereby promoting Akt phosphorylation and NF-κB release, as well as cell survival, to play neuroprotective roles [92]. Changes in the expression of writers such as METTL14, WTAP, and RBM15 may not exist in the rat MCAO model [57].

In mouse MCAO and OGD-induced microglia models, YTHDF1 expression was upregulated, owing to the downregulation of miR-421-3p expression, and YTHDF1 then recognized the m6A-modified p65 mRNA and promoted its translation and nuclear transport, finally activating the NF-κB signaling pathway and promoting cellular inflammation [55]. Another study in a mouse MCAO model supported the trend that YTHDF1 and YTHDF3 expression was upregulated, while FTO expression was downregulated, especially in neurons, with increased global m6A levels in the peri-infarct cortex [56]; meanwhile, gene ontology (GO) analysis of differentially expressed mRNAs showed their relationship with inflammation, apoptosis, and transcriptional regulation [93] (Table 1). All the above studies indicate that m6A writers, erasers, and readers of glial cells may participate in the occurrence and development of ischemic brain injury.

### 3.4. AD Mediated by RNA m6A-Modified Glial Cells

AD is the most common neurodegenerative disease, with extracellular amyloid-β (Aβ) plaque formation and intracellular neurofibrillary tangles of tau protein deposition, which are related to functional changes in glial cells. Degenerative cognitive impairment is associated with alterations in OLs and myelin, including a decrease in number, changes in shape or phenotype, and myelin damage in the preclinical stage [94]. Microglia preferentially express AD risk factors (e.g., triggering receptor expressed on myeloid cells 2 (TREM2) and P2RX7) and release inflammatory factors, triggering the classical complement cascade that damages neurons and promotes Aβ deposition. Moreover, excessive Aβ levels disrupt the balance between microglial phagocytosis and Aβ production [95]. As for astrocytes, EAAT and astrocyte-neuron lactate shuttle are impaired, leading to the production and accumulation of Aβ and tau protein under the stimulation of cytokines, such as TGF-β and IL-1β, while the risk gene *ApoE4* regulates cerebrovascular functions, resulting in neuronal energy metabolism dysfunction, glutamate excitatory toxicity, and the development of AD [96].

Recent studies have shown that APOE4 is closely related to the RNA m6A regulatory proteins METTL3, METTL16, and YTHDC2. Meanwhile, RNA-seq expression profiles showed that the brain FTO, YTHDC2, and YTHDF2 were the most differentially expressed among the different cognitive groups of AD, with the lowest expression levels in AD patients [58]. METTL3 expression was significantly downregulated in the middle temporal gyrus associated with early cognitive impairment in AD patients, suggesting a key role of METTL3 in early AD [61], while METTL3 knockdown in hippocampal pyramidal neurons increased oxidative stress and abnormal cell cycle events, leading to significant reductions in the number of NeuN^+^ neurons and synapses, the activation of caspase-9/3, and increased apoptosis, causing cognitive deficits [61]. In another study on AD patients, METTL3 expression was downregulated and RBM15B expression was upregulated in the hippocampus, while METTL3 was significantly accumulated in insoluble tau protein, suggesting a correlation between METTL3 and tau protein deposition [59]. In addition, IGF2BP2 was highly expressed in the entorhinal cortex, hippocampus, posterior central gyrus, and superior frontal gyrus in AD patients and was significantly enriched in extracellular matrix receptor interaction, focal adhesion, cytokine–cytokine receptor interaction, and the TGF-β signaling pathway, which might be related to the occurrence of AD [60]. In addition, because synaptic plasticity is the basis of cognitive function, ALKBH5, a typical eraser of RNA m6A modification, is involved in short-term synaptic plasticity and postsynaptic local ribosomal translation [97]. These findings suggest that the RNA m6A epigenetic modification mechanism may be involved in the regulation of AD occurrence and development.

The correlation between AD and m6A modification of RNA in glial cells has been reported (Table 1). For example, autopsy results of AD patients showed a significant increase in m6A levels in astrocytes and microglia in the hippocampus, although immunohistochemistry tests did not show significant changes in the expression levels of m6A-related proteins (including METTL3, METTL14, WTAP, FTO, and YTHDF1), similar to those in hippocampal pyramidal neurons. However, microglia and astrocytes in the hippocampus were still activated after METTL3 knockdown, indicating that although METTL3 was less expressed in astrocytes and microglia, it still had some activity, suggesting that METTL3 may play a role in regulating the function of glial cells [61]. In addition, the expression of FTO and YTHDF1 in astrocytes was significantly upregulated, as reported in studies on streptozotocin-induced AD models. When MO-I-500 was used as an FTO inhibitor to downregulate the expression of FTO in astrocytes, the survival rate increased significantly, oxidative stress and cell apoptosis were significantly reduced, and mitochondrial dysfunction and energy metabolism disorder were significantly improved, exhibiting the toxic effect of FTO and the nerve protective effect of its inhibitor in AD [62]. However, astrocyte-derived IL-1β vesicles increased the binding of the m6A reader hnRNPC to amyloid precursor protein (APP) mRNA, to promote APP translation and Aβ production, thereby exacerbating chronic inflammation in AD [63]. In conclusion, m6A modifications related to glial cells in AD are mostly concentrated in astrocytes, and a more detailed regulatory mechanism is unknown. Therefore, more studies are needed to explore the modes of action and mechanisms of m6A modification.

### 3.5. PD Mediated by RNA m6A-Modified Glial Cells

PD is a delayed neurodegenerative disease, characterized by bradykinesia and quiescent tremor, and is associated with misfolded α-synuclein (SNCA) assembly into fibrous inclusions and loss of dopamine (DA) neurons in the substantia nigra of the midbrain. As the striatum is important for dopamine transmission, impaired striatal function often accompanies PD [98]. As a DAMP, SNCA activates microglia via TLR2 and the downstream NF-κB pathway with the NLRP3 inflammasome, which releases IL-1β and engulfs SNCA. While there is deposition of SNCA in astrocytes, resulting in their dysfunction in promoting the survival of DA neurons and maintaining the stability of BBB. Moreover, with C1q and other factors released by microglia, astrocytes release TNF-α, IL-1β, and other neurotoxic factors, to aggravate neuronal injury [99]. Although we have a certain understanding of the pathology of PD, the analysis of the deeper level of signal pathway regulation is still insufficient. 

Recent basic studies have suggested a correlation between PD and RNA m6A modification. For example, the loss of METTL14 affects the excitability of neurons in the striatum, corresponding to learning and memory functions, and increases the sensitivity to dopamine drugs [100]. However, in the 6-OHDA-induced PD rat model, only the m6A level in the striatum was decreased, with significantly upregulated ALKBH5 expression and no change in FTO expression, while the m6A level in the midbrain was not significantly changed with upregulated FTO expression, in line with the PD neuron model, indicating that FTO may be transmitted through axons and mediate the demethylation of m6A-modified RNA in the striatum. FTO then activates NMDAR1 in an m6A-dependent manner, resulting in increased oxidative stress, Ca^2+^ influx and overload, impaired mitochondrial function, and promotion of apoptosis, indicating the potential role of FTO in the death of DA neurons [64]. At the same time, because excessive Mn accumulation in the striatum can induce increased muscle tone, tremors, and other symptoms similar to those of PD, the expression of FTO was downregulated by the upstream transcription factor Foxo3a, thus leading to a decreased demethylation of epinephrine-B2 mRNA and an increase in its m6A level. The m6A-modified epinephrin-B2 mRNA was finally recognized and degraded by YTHDF2, affecting normal axonal conduction and leading to damage to DA neurons and motor disorders [65]. In addition, FTO is associated with the activation of G protein-gated inwardly rectifying potassium (GIRK) channels mediated by DA receptors (D2R and D3R) in DA neurons, which play a role in a variety of behaviors and diseases (e.g., schizophrenia) [101]. Inhibition of FTO could effectively promote the survival of DA neurons [102], suggesting that FTO might be related to rewarding behaviors, such as feeding, and may also provide some references for the occurrence of PD. The expression of RNA m6A reader hnRNPC was significantly downregulated in PD, and its overexpression promoted the proliferation of PC12 cells and inhibited the expression of IFN-β, IL-6, and TNF-α, without significant effects on autophagy [103]. This result suggests that hnRNPC may promote apoptosis and cause immune inflammation by inhibiting the proliferation of DA neurons, leading to the occurrence and development of PD (Table 1).

### 3.6. Neuropsychiatric Disorders Mediated by RNA m6A-Modified Glial Cells

Depression is a serious neuropsychiatric disorder that mainly manifests as low mood, loss of interest, self-blame, pessimism, and corresponding physical symptoms [104]. Although it is generally considered to be a result of neuronal lesions, the discovery that glial cell-specific proteins such as P2RX7, S100 calcium binding protein B (S100B), and GFAP, are involved in the occurrence and progression of depression, especially in those major depression disorder (MDD) patients presenting with suicidal behaviors, as well as the alterative crosstalk of glial cells-neurons exacerbating cell damage, suggests that glial cells play unique roles in specific areas [105,106,107].

A recent analysis showed that there was an RNA m6A eraser ALKBH5 mutation in Chinese Han people with MDD [108], while the rs9939609 A mutation in the FTO gene was negatively correlated with depression [109]. The m6A and N6, 2′-O-dimethyladenosine (m6A/m) levels in the peripheral blood of MDD patients stimulated by glucocorticoids were significantly reduced [67], suggesting that RNA m6A/m modification is associated with MDD. In addition, studies have also shown that the circSTAG1 level is decreased in the peripheral blood of MDD patients and hippocampus of mice with stress-induced depression (CUS), especially in the astrocyte cytoplasm. Overexpression of circSTAG1 blocked the translocation of ALKBH5 from the cytoplasm to the nucleus, resulting in the upregulation of m6A modification of the fatty acid amide hydrolase (FAAH) mRNA 3′ UTR and degradation of its stability, which attenuated the astrocyte dysfunction and depressive-like behaviors of CUS mice [66], indicating that nuclear ALKBH5 promotes the progression of depression. In addition, time-dependent m6A/m level changes in synaptic and neuronal function-related mRNAs in the prefrontal cortex (PFC) and basolateral and central amygdala (AMY) were found in an acute stress-induced depression model, especially in the 5′ UTR and near the stop codon, which might be regulated by the m6A reader FMRP. In addition, the expression of RNA m6A writer METTL3 was significantly downregulated in the PFC and AMY, while the expression of erasers FTO and ALKBH5 was regionally upregulated in the PFC with decreased m6A/m levels, and their expression was downregulated in the AMY with increased m6A/m levels. At the same time, the study also showed that after METTL3 or FTO deletion, excitatory neurons were depleted, fear memory and spontaneous mining behaviors were increased, and long-term potentiation (related to memory and synaptic plasticity) was decreased in mice [67], indicating that m6A modification might be related to depression. However, in mice with chronic constraint stress (CRS)-induced depression, the expression of FTO was downregulated significantly in the hippocampus, and interaction with calcium-calmodulin-dependent protein kinase II (CaMKII)/cAMP response element binding protein (CREB) resulted in a reduction of dendritic synaptic structures and the expression of the synapse plasticity molecules synaptophysin and postsynaptic density protein 95 (PSD95), which may be associated with cognitive impairment [68]. Another study showed that FTO expression was also downregulated in the hippocampus of MDD patients and in three mouse models of depression, including that induced with CRS, which may regulate adrenoceptor β2 (ADRB2, regulating synaptic plasticity and depressive behaviors) and activate downstream c-MYC/SIRT1 to reverse depression-like behaviors [69]. In addition, studies on tricyclic antidepressants that may not depend on the monoamine mechanism showed that after drug treatment, the expression of FTO in the ventral tegmental area (VTA, controlling diets and probably associated with weight gain induced by antidepressants) was upregulated significantly, whereas there were no significant changes in METTL3, METTL14, and ALKBH5 expression, leading to a significant reduction of the m6A level. Furthermore, it was possible to relieve depression-like behaviors and increase feeding in mice, by inhibiting the gene transcription of the downstream stress-sensitive neuropeptide Cartpt (which affects mood through neurotransmitter receptors and other factors) and Ucn (which acts on corticotrophin-releasing factor receptors to induce and control stress responses) [70]. These findings suggest that FTO is associated with synaptic damage and cognitive impairment in depression and imply a potential association between FTO and other diseases involving myelin injuries (Table 1).

### 3.7. Neurodevelopmental Disorders Mediated by RNA m6A-Modified Glial Cells

Cognitive impairment in schizophrenia (SCZ) is a neurodevelopmental disorder characterized by positive and negative symptoms, accompanied by social dysfunction. SCZ is associated with myelinogenesis defects and white matter damage, and the number of OLs and myelination are significantly reduced in the PFC and hippocampus [110,111]. Microglia are activated, to perform synapsis pruning and phagocytosis of broken neurons, and inhibit the release of neurotrophic factors, to induce the death of gray matter neurons [112]. The autopsy results of some SCZ patients showed increased astrocytes, which might be associated with neurotransmitter response, synaptic dysfunction, and inflammatory response [113,114].

In studies of cerebral cortex development in mice, the RNA m6A regulators METTL3 and FTO were found to promote NSC proliferation and neuronal differentiation through the BDNF/Akt pathway, which is associated with learning and memory [115]. YTHDF2 deficiency could lead to embryo death [116], suggesting that it is related to neural development, while METTL14 or METTL3 deficiency could prolong the cell cycle of radial glial cells and cortical neurogenesis in the postpartum stage, indicating that m6A modification plays an important role in maintaining the rapid transformation between NSC proliferation and differentiation and in facilitating the normal process of neurogenesis [117]. In addition, co-location analysis of genome-wide association study (GWAS) and m6A quantitative trait loci (QTL, which can also be searched for single nucleotide polymorphism (SNP)) showed that SCZ-related rs7285557 downregulated the expression of the long intergene non-coding RNA Linc00634, which reflected the m6A epigenetic regulation of SCZ [118]. Meanwhile, the pathogenesis-related genes of SCZ, including ZSCAN12, HLA-DQA1, and SNX19, were identified by including an m6A database and methylation quantitative trait loci (meQTL) in GWAS, mainly involving antigen presentation and processing [119], suggesting that more RNA m6A modification sites may be found in SCZ in the future and predicted by GWAS.

### 3.8. GBM Mediated by RNA m6A-Modified Glial Cells

GBM is astrocytoma with WHO grade IV and is the most common and malignant primary brain tumor in adults. Owing to its prominent heterogeneity, surgical resection combined with radiotherapy and/or chemotherapy may cause recurrence and temozolomide resistance [120]. GBM is driven by glioblastoma stem cells (GSCs), in which the tumor microenvironment regulates homeostasis and promotes GSC proliferation, survival, immune escape, and invasion of non-tumor cells, and secretes molecules and extracellular matrix (ECM). Glial cells interact with GSCs to promote the occurrence and development of GBM. For example, tumor-associated astrocytes with reactive hypertrophy and hyperplasia secrete tumor growth factors, the gap junction protein Cx43, and matrix metalloproteinases (MMPs), to reduce apoptosis, promote angiogenesis and ECM degradation, and enhance tumor invasion and migration [121]. Glioma-associated microglia/macrophages, which are positively correlated with the number of GBM cells, not only secrete TGF-β, IL-6, IL-1β, MMPs, and VEGFA to promote cell proliferation and edema, as well as degrade ECM, but are also polarized into immunosuppressive phenotypes to inhibit T cell proliferation and promote tumor progression [122].

Based on the cancer genome map, the Cancer Genome Atlas (TCGA) and Chinese Glioma Genome Atlas (CGGA) databases of GBM patients, and genotype-tissue expression (GTEx) databases of healthy people, m6A-modified subgroups and differentially expressed gene groups, with clinical characteristic and biological pathway differences, were screened using a principal component analysis, and a m6A score model was constructed [123]. In addition, the correlation between m6A regulators and clinical pathological features and molecular phenotypes subjected to cluster analysis was evaluated, to establish a Cox regression model of risk genes, which could predict the immune cell infiltration degree, chemotherapy and immunotherapy effects, and clinical prognosis, as well as guide the treatment of GBM patients to a certain extent [124] (Table 1).

Many studies have revealed that m6A regulators can mediate the occurrence and development of GBM in various ways (Table 2), especially METTL3. On one hand, METTL3 could affect the expression of the oncogene ADAM, to inhibit proliferation [71]. On the other hand, further studies indicated that METTL3 can promote the progression of GBM by regulating epithelial–mesenchymal transition and vascular patterns, RNA editing, and mRNA decay, to accelerate the cell cycle or enhance the resistance to radiotherapy and chemotherapy, by promoting DNA repair [72,73,125]. Other m6A regulators have roles that are similar to those of METTL3. In general, although there are differences in the expression levels of certain m6A regulators in different studies, varied trends always reflect tumor progression and the enhancement of chemoradiotherapy resistance.

## 4. Discussion

m6A is a critical method of RNA modification at the post-transcriptional and translational levels. It is characterized by alteration of gene expression through RNA stability, splicing, nuclear export, translation, or decay, thereby affecting disease phenotypes. Current research on the correlation between m6A modification and neurological diseases is mostly focused on the neuronal background. For example, studies on TBI and ischemic brain injury are mostly focused on cortical neurons, while those on PD concentrate on DA neurons. More attention has been paid to the association of total m6A levels and their regulators with the neuronal phenotypic changes in specific areas. It can be inferred from the existing studies that m6A modification can mediate neuronal death or damage from apoptosis, inflammation, energy metabolism disruption, oxidative stress, and other aspects; thus, the occurrence and development of neurological diseases are promoted. The levels of m6A and their regulators can be used as potential biomarkers for diseases. For example, PMS can be distinguished from RRMS through the m6A levels in CSF, and the treatment effects and clinical prognosis of GBM can be predicted by screening and scoring m6A levels. Changes in the CNS are closely related to glial cells, particularly microenvironmental fluctuations and myelin sheath injuries. However, few studies have explored the relationship between m6A modification and neurological diseases in the context of glial cells. Moreover, previous studies have not refined the m6A modification of specific genes in neurons or glial cells, but broadly in certain regions; the function of m6A modification in neuron–neuron and glial cells–neurons crosstalk is not clear. Glial cells, as important regulators for CNS homeostasis and indispensable components of cell interactions, have essential and important functional connections for neurons or themselves. Furthermore, changes in their phenotypes and functions also occur in neurological diseases. For example, the most significant pathological changes are dysfunction of the OL lineage in demyelination diseases. The present study merely illuminates that Prrc2a and METTL14 are related to myelin development, and FTO has potential correlations with axonal injury, which does not directly clarify changes in the expression of these proteins and their possible roles in pathological states. In addition, as microglia and astrocytes are the main participants of neuroinflammation in neurodegenerative diseases, there have been several studies on the pro-inflammatory role of astrocytes and their functions in energy metabolism in AD models, while the relationships between m6A modification and glial cells are unknown.

Research on the correlation between RNA m6A modifications and neurological diseases illustrated that m6A modification may reduce mRNA stability under normal circumstances. The feedback mechanism under normal physiological conditions can be dynamically adjusted. However, under disease states, negative feedback effects are heightened, and damage and protection effects exist at the same time. For example, the expression of writers, erasers, and readers is upregulated in acute injury, cancer, and other proliferative pathological states, reflecting an extreme disturbance of the intracellular environment. However, in chronic stress diseases, such as neurodegenerative and neuropsychiatric disorders, the overall level of m6A is mostly decreased. For instance, the expression of METTL3, the most classic m6A methyltransferase, is frequently upregulated in the early stages of diseases, rather than that of other erasers and readers, and this change is more significant in acute disease. Although METTL3 expression might be downregulated owing to the negative feedback in the later stages of the disease, the primary role of METTL3 and the follow-up interactions with other RBPs are still indicated. It is worth noting that the expression of FTO was upregulated or downregulated in all of the above CNS diseases; however, even if the upregulated FTO expression changes the levels of same downstream proteins, the trends of changes in the protein levels could be opposite, suggesting the existence of different intermediate activation pathways. The differences between in vitro and in vivo experiments are not only due to genetic heterogeneity but also associated with the feedback mechanism of the CNS in vivo. Mechanistic exploration should not be limited to the superficial data on correlations between changes in m6A regulators and typical proteins in corresponding pathways, as the statistical correlations between these two do not indicate the existence of an inevitable causality. RNA immunoprecipitation (RIP), RNA pull-down, and other technologies should be employed to discover new interacting proteins. In addition, changes in FTO expression in various diseases suggest a possible link between lipid metabolism and neurological diseases. As FTO is more likely to bind to m6Am, which can increase RNA stability, compared with that under m6A, the relationship between METTL3 and FTO cannot be simply regarded as cooperative or antagonistic. Second, studies on RNA m6A modification in glial cell tumors are also abundant, mainly illustrating that the expression of m6A regulators is significantly upregulated in GBM, such as hnRNPC, ALKBH5, and IGF2BP, suggesting that abnormal m6A modifications occur when glial cells undergo unrestricted proliferation.

The present studies on RNA m6A regulators in neurological diseases have yielded different results, even on the expression of the same protein in specific diseases or different models of the same disease. Although such contradictory results might be due to the differences in the levels of functional readers and genetic heterogeneity, more studies with novel methods and techniques are still needed, to further confirm these findings from various aspects. Additionally, the current studies are mostly limited to the use of certain protein inhibitors. In addition to pharmacological inhibitors of FTO, there are no proven inhibitors of other m6A regulators; thus, the effects of target proteins in diseases are not clear. The use of cycloleucine for RNA transcription termination to determine the regulatory mechanism of readers at the post-transcriptional level is also an indirect proof of the roles of m6A, which requires structural analysis or more accurate analytical methods for confirmation. When gene expression is upregulated, the current studies generally used shRNA or siRNA for gene knockdown, to explore the subsequent mechanism; however, RNA interference is less efficient and accurate than gene editing. Therefore, the accuracy of the results is questionable. At the same time, the m6A-seq/MeRIP used in most studies to analyze the m6A modification sites of RNA was not as accurate as miCLIP to a single base; the existing results were drawn on the premise of confirming m6A regulators with their binding sequences in the early stage and could not distinguish m6A from m6Am [134]. Therefore, questions remain, such as whether the existing or later discovered RBPs are indeed m6A regulators. Although there is a distinction, that m6Am is mostly distributed in the 5′ cap and m6A is more likely to be distributed in the 3′ UTR, certain speculations can be drawn based on the binding regions of RBPs; however, they are still not accurate. Therefore, establishing disease models of glial cell-specific m6A-related gene knockout or overexpression vectors and using more accurate m6A analysis techniques can better clarify the role of the RNA methylation of glial cells in specific diseases, further improve the pathogenesis of diseases, and identify potential therapeutic targets. Colocalization of m6A QTL and GWAS associations can, not only verify the interaction between the discovered RBPs and possibly mRNA, but also identify potential undiscovered RBPs. m6A modification sites may possibly be predicted at the post-transcriptional level by examining SNP changes in diseases through GWAS. The accuracy of single bases also helps identify RBP-binding sequences and better address the specificity of the recognition of m6A sites combined with RNA structures.

## 5. Conclusions

In conclusion, m6A modification of glial cells is indispensable to the occurrence and development of neurological diseases, especially Alzheimer’s disease and glioblastoma, although most research has focused on astrocytes, regarding the aspects of apoptosis or oxidative stress, and studies on schizophrenia or oligodendrocyte lineage cells are rare. These complex mechanisms need to be better understood using combined analysis of upstream and downstream targets and with more advanced techniques, in future directions for clinicians and researchers.

## Figures and Tables

**Table 1:** Roles of RNA m6A-modified glial cells in neurological diseases.

Neurological Diseases	Glial Cells	Changes in RNA m6A Regulators		Downstream RNA or Pathways	Citation
TBI	microglia, astrocytes	METTL14, FTO	downregulated	Bcl-2 downregulated, Dll4 and CD14 upregulated	[49]
		METTL3, WTAP, VIRMA, ALKBH5	no changes		
MS	OLs	METTL14	-	neurofascin 155	[51]
		hnRNPA2/B1		MBP	[52]
		Prrc2a		Olig2	[53]
		FTO		GAP-43	[54]
Ischemia brain injury	microglia	YTHDF1	upregulated	p65	[55]
	microglia, astrocytes	YTHDF1, YTHDF3	upregulated	-	[56]
		FTO	downregulated	-	[57]
AD	microglia, astrocytes	METTL3, METTL16	upregulated	APOE4	[58]
		YTHDC2	downregulated		
		METTL3	downregulated	tau protein	[59]
		RBM15B	upregulated	-	
		IGF2BP2	upregulated	transcripts related to extracellular matrix receptor interaction, focal adhesion, cytokine-cytokine receptor interaction, and TGF-β signaling pathways	[60]
		METTL3, METTL14, WTAP, FTO, YTHDF1	no changes	-	[61]
	astrocytes	FTO, YTHDF1	upregulated	transcripts related to oxidative stress, apoptosis, and mitochondrial functions	[62]
		hnRNPC	-	APP	[63]
PD	microglia, astrocytes	ALKBH5, FTO	upregulated	NMDAR	[64]
		FTO	downregulated	epinephrine-B2	[65]
Depression	astrocytes	ALKBH5	upregulated	FAAH	[66]
	microglia, astrocytes	METTL3	downregulated	transcripts involved in stress response and synaptic plasticity	[67]
		FTO, ALKBH5	upregulated or downregulated		
		FTO	downregulated	CaMKⅡ/CREB	[68]
				β2-adrenergic receptor	[69]
			upregulated	Cartpt and Ucn	[70]
GBM	astrocytes, microglia	METTL3	downregulated	ADAM 19	[71]
			upregulated	SRSF	[72]
			downregulated	transcripts related to epithelial–mesenchymal transition and vasculogenic mimicry	[73]
			upregulated	SOX2	[74]
		METTL14	downregulated	ADAM 19	[71]
		FTO	upregulated	CLIP3	[75]
		ALKBH5	upregulated	FOXM1	[76]
			upregulated	transcripts related to epithelial–mesenchymal transition and vasculogenic mimicry	[73]
			upregulated	YAP1	[77]
		YTHDF2	upregulated	MYC, VEGF	[78]
		IGF2BP2	upregulated	OIP5-AS1, miR-129-5p	[79]
			upregulated	lncRNA CASC9	[80]
		IGF2BP3	upregulated	-	[81]

**Table 2:** m6A regulators and their functions in GBM.

m6A Regulators	Cell Lines or Tissues	Expression Level	m6A Alteration	Functions	Citation
METTL3	tumor tissues from GBM patients	↓	m6A decreased	METTL3 knockdown enhanced GSC growth and self-renewal, as well as GBM progression probably via ADAM metallopeptidase domain 19 (ADAM, oncogene).	[71]
	surgical specimens from GBM patients, subcutaneous tumor model and intracranial GBM xenograft model and U251 and U87MG cell lines	↑	m6A increased	METTL3/YTHDC1-dependent SRSF mRNA nonsense-mediated decay increased alternative splicing of Bcl-x and nuclear receptor corepressor 2 (maintain cell specificity and tissue homeostasis) and GBM development and proliferation.	[72]
	primary tumor GSC, MGG8	unknown	m6A modification mainly in the 3′ UTR	METTL3-dependent m6A modification regulated RNA editing and Notch pathway stimulation in GSCs.	[125]
	U138MG, T98G, A172, U118MG, U87MG, and LN18	↑	m6A increased near the stop codon	Adenosine-to-inosine RNA editing catalyzing protein enhanced by METTL3/YTHDF1 bound with the cyclin-dependent kinase 2 transcript to accelerate cell cycle and control cell proliferation and tumor growth.	[126]
	tumor tissues from GBM patients and U87MG cell line	↓	m6A decreased	Knockdown of METTL3 regulated epithelial–mesenchymal transition (EMT) and vasculogenic mimicry (VM) processes with decreased survival time.	[73]
	U87MG and U251 cell lines exposed to temozolomide (TMZ) and subcutaneous glioma xenograft model	↑	m6A increased	METTL3 promoted the TMZ resistance of GBM cells by increasing DNA repair protein MGMT and ANPG levels in an m6A-dependent manner.	[127]
	tumor tissues from GBM patients	↑	m6A increased	METTL3 methylated SOX2 mRNA, which recruited human antigen R (HuR) to the modified SOX2 and stabilized the mRNA to enhance GSC self-renewal and dedifferentiation and increase DNA repair for radioresistance.	[74]
	U87MG and U251 cell lines	↑	no change	RNA-binding protein NKAP combined SLC7A11, a ferroptosis defense protein, recruited the splicing factor proline and glutamine-rich to recognize the splice site, and conducted TTS splicing event on SLC7A11 transcript and the retention of the last exon, to protect GBM cells from ferroptosis.	[128]
METTL14	tumor tissues from GBM patients	↓	m6A decreased	METTL14 knockdown enhanced GSC growth and self-renewal and GBM progression probably via ADAM.	[71]
FTO	tumor tissues from GBM patients	↑	m6A decreased	miR-145 in differentiated glioma cells (DGCs) mediated the formation of FTO/AGO1/ILF3/miR-145 complexes on clinically relevant tumor suppressor gene (CLIP3) and promoted CLIP3 demethylation by FTO and nascent translation to induce the transformation of DGCs and GSCs.	[75]
	TS576, GBM-GSC-23, and GBM-6 cell lines	↑	-	FTO inhibitors impaired self-renewal in GSCs.	[71]
	U251, LN229, U87, SHG-44, and LN18 cell lines	↑	-	lncRNA just proximal to the X- inactive specific transcript modulated and stabilized PDK1 in an FTO-dependent manner to promote aerobic glycolysis and TMZ chemoresistance.	[129]
ALKBH5	GBM xenografts and surgical specimen tissue slides	↑	m6A decreased in FOXM1 pre-mRNA	ALKBH5 demethylated forkhead box protein M1(FOXM1) and affected HuR association with FOXM1 nascent transcripts in GSCs, leading to cell proliferation and tumor growth.	[76]
	U87 and GL261 cell lines	↑	most m6A-modified transcripts downregulated	ALKBH5 stabilized lncRNA NEAT1 by demethylation; therefore, NEAT1 controlled paraspeckle assembly and SFPQ relocation from *CXCL8* promoter and facilitated TAM recruitment and immunosuppression through CXCL8/IL8.	[130]
	GBM biopsy specimens	↑	-	ALKBH5 regulated GBM invasion through YAP1 expression and increased radioresistance by regulating homologous recombination and DNA-damage repair.	[77]
	tumor tissues from GBM patients and U87MG cell line	↑	m6A decreased	ALKBH5 overexpression causes a highly scattered pattern of cytoskeleton through the rearrangement of F-actin to enhance the EMT and VM process and growth of GBM cells.	[73]
YTHDF2	surgical resection samples from patients	↑	No change in m6A peaks, but in m6A distribution	YTHDF2 stabilized MYC and VEGF transcripts in an m6A-dependent manner following upregulated expression of IGFBP3 to promote tumor growth.	[78]
	Hs683, SW1783, T98G, U87MG, LN299 cell lines, and animals injected with GSC or LN299 cells	↑	-	Sustained by EGFR/SRC/ERK signaling, YTHDF2 downregulated LXRα and HIVEP2 through m6A-dependent mRNA decay and inhibited LXRα-dependent cholesterol homeostasis, to promote tumorigenesis.	[131]
	CGGA website and TCGA database, and H4, LN299, and U87 cell lines	↑	m6A decreased in UBXN1 mRNA	YTHDF2 accelerated UBXN1 mRNA decay by recognizing the m6A modification mediated by METTL3 and enhanced NF-κB activation, to promote GBM progression.	[132]
IGF2BP2	U251 cell line, surgical tissue samples, and mouse xenograft model	↑	-	IGF2BP2 induced GBM cell chemoresistance by downregulating forkhead box protein O1-mediated phosphotyrosine interaction domain containing 1 expression via stabilizing lncRNA DANCR.	[133]
	GBM surgical specimens and U251, U87, A172, and SHG44 cell lines	↑	-	IGF2BP2 regulated by the sponge interaction between OIP5-AS1 and miR-129-5p promoted cell chemoresistance to TMZ and cell growth in GBM.	[79]
	GBM surgical specimens and U87MG and U251 cell lines	↑	m6A peaks in 5′ UTR and 3′ UTR	IGF2BP2 stabilized lncRNA CASC9 to accelerate the aerobic glycosis of GBM by enhancing hexokinase 2 mRNA stability.	[80]
IGF2BP3	GBM specimens	↑	-	Upregulated IGF2BP3 expression was associated with poor OS and prognosis.	[81]

## Data Availability

Not applicable.
